# Feasibility of Using Convalescent Plasma Immunotherapy for MERS-CoV Infection, Saudi Arabia

**DOI:** 10.3201/eid2209.151164

**Published:** 2016-09

**Authors:** Yaseen M. Arabi, Ali H. Hajeer, Thomas Luke, Kanakatte Raviprakash, Hanan Balkhy, Sameera Johani, Abdulaziz Al-Dawood, Saad Al-Qahtani, Awad Al-Omari, Fahad Al-Hameed, Frederick G. Hayden, Robert Fowler, Abderrezak Bouchama, Nahoko Shindo, Khalid Al-Khairy, Gail Carson, Yusri Taha, Musharaf Sadat, Mashail Alahmadi

**Affiliations:** King Saud bin Abdulaziz University for Health Sciences, King Abdullah International Medical Research Center, Riyadh, Saudi Arabia (Y.M. Arabi, A.H. Hajeer , H. Balkhy, S. Johani, A. Al-Dawood, S. Al-Qahtani, A. Bouchama, K. Al-Khairy, M. Sadat, M. Alahmadi);; Naval Medical Research Center, Silver Spring, Maryland, USA (T. Luke, K. Raviprakash);; Alfaisal University, Riyadh (A. Al-Omari);; King Saud bin Abdulaziz University for Health Sciences, Jeddah, Saudi Arabia (F. Al-Hameed);; University of Virginia School of Medicine, Charlottesville, Virginia, USA (F.G. Hayden);; University of Toronto, Toronto, Ontario, Canada (R. Fowler);; World Health Organization, Geneva, Switzerland (N. Shindo);; University of Oxford Centre for Tropical Medicine, Oxford, UK (G. Carson);; King Abdulaziz Medical City, Al-Ahsa, Saudi Arabia (Y. Taha)

**Keywords:** Middle East respiratory syndrome coronavirus, MERS-CoV, Middle East respiratory syndrome, MERS, intensive care, convalescent phase, convalescent plasma, convalescent-phase plasma, feasibility study, immunotherapy, seroreactive, neutralizing antibodies, antibody titers, viruses, ELISA, microneutralization, indirect immunofluorescent antibody assay, IFA, Saudi Arabia, humans, respiratory infections

## Abstract

We explored the feasibility of collecting convalescent plasma for passive immunotherapy of Middle East respiratory syndrome coronavirus (MERS-CoV) infection by using ELISA to screen serum samples from 443 potential plasma donors: 196 patients with suspected or laboratory-confirmed MERS-CoV infection, 230 healthcare workers, and 17 household contacts exposed to MERS-CoV. ELISA-reactive samples were further tested by indirect fluorescent antibody and microneutralization assays. Of the 443 tested samples, 12 (2.7%) had a reactive ELISA result, and 9 of the 12 had reactive indirect fluorescent antibody and microneutralization assay titers. Undertaking clinical trials of convalescent plasma for passive immunotherapy of MERS-CoV infection may be feasible, but such trials would be challenging because of the small pool of potential donors with sufficiently high antibody titers. Alternative strategies to identify convalescent plasma donors with adequate antibody titers should be explored, including the sampling of serum from patients with more severe disease and sampling at earlier points during illness.

## Abstract 2

Efficacy testing will be challenging because of the small pool of donors with sufficiently high antibody titers.

Middle East respiratory syndrome coronavirus (MERS-CoV) was initially identified in September 2012 when a patient in Saudi Arabia with a severe, acute respiratory infection and acute renal failure died (*1*). As of June 19, 2016, more than 1,733 MERS-CoV cases and at least 628 associated deaths had been identified; >80% of the cases occurred in Saudi Arabia (*2*). More than 20 countries outside of the Arabian Peninsula have reported MERS-CoV cases, and the 2015 outbreak in South Korea with attendant mortality has reinforced concerns about international outbreaks (*3*). No specific treatment has been proven effective for MERS-CoV infection.

Convalescent plasma containing MERS-CoV–specific antibodies from recovered patients has been suggested as a potential therapy for infected persons (*4*). Convalescent plasma has been used to treat several other viral infections, including those caused by the severe acute respiratory syndrome coronavirus (SARS-CoV), avian influenza A(H5N1) virus, and influenza A(H1N1)pdm09 virus (*5*–*10*). A recent metaanalysis of studies using passive immunotherapy for treatment of severe acute respiratory infections of viral etiology suggests that the timely use of convalescent blood products, particularly those with neutralizing antibodies, results in a reduced death rate (*11*). Public Health England and ISARIC (the International Severe Acute Respiratory and Emerging Infection Consortium) published a decision-making support tool on potential therapies for MERS-CoV that highlights convalescent plasma and other neutralizing antibody–containing immunotherapeutics (e.g., hyperimmune immunoglobulins and monoclonal antibodies) as the most promising potential treatments for serious MERS-CoV illness and deserving of evaluation in human clinical trial(s) (*4*).

However, no data support the feasibility of obtaining convalescent plasma from patients who have been exposed to MERS-CoV or recovered from infection with the virus. Camels are the likely source for most animal-to-human transmission and appear to have long-lasting antibody responses; in preclinical models, such antibodies appear effective in reducing the severity of pathologic changes in infected lungs (*12*). However, the antibody response to MERS-CoV infection in humans is poorly defined. Thus, we planned a 2-phase study to 1) determine the feasibility of collecting high-titer convalescent plasma from MERS-CoV patients and contacts and, if successful, to 2) conduct a pilot therapeutic study using convalescent plasma in symptomatic MERS-CoV patients with moderate to severe illness. Herein, we report on the feasibility study.

## Methods

In collaboration with the King Abdullah International Medical Research Center, the Gulf Cooperation Council Infection Control Center, and the World Health Organization (WHO)–International Severe Acute Respiratory and Emerging Infection Consortium MERS-CoV Working Group, we developed a study protocol to screen potential donors, collect high-titer convalescent plasma, and administer the plasma in a clinical trial (*13*). The study was approved by the Ministry of the National Guard Health Affairs Institutional Review Board (approval no. IRBC/233/14, June 9, 2014) and registered in ClinicalTrials.gov (NCT02190799). We conducted the study at King Abdulaziz Medical City, a 1,100-bed tertiary care center in Riyadh, Saudi Arabia. The hospital is accredited by the Joint Commission International, and the hospital’s Department of Pathology and Laboratory Medicine is accredited by the College of American Pathologists and the American Association of Blood Banks.

### Study Population

We screened potential convalescent plasma donors from 3 cohorts: 1) patients with acute respiratory illness who were suspected of having MERS-CoV or who were confirmed MERS-CoV–positive by real-time reverse transcription PCR (rRT-PCR) of upper or lower respiratory secretions; 2) healthcare workers exposed to a laboratory-confirmed MERS-CoV patient, as identified by ongoing active surveillance of the hospital Infection Prevention and Control Department; and 3) household contacts of patients with laboratory-confirmed MERS-CoV infection. We obtained written informed consent for MERS-CoV serologic testing from all healthcare workers and household contacts. Medical teams ordered serologic testing as part of the clinical care for patients with suspected or confirmed MERS-CoV infection; no additional informed consent was required. Healthcare workers completed a self-administered survey that asked questions about the nature, duration, and degree of exposure to patients with laboratory-confirmed MERS-CoV infection. For all study participants, we documented the time that had elapsed from symptom onset or exposure to the collection of samples for testing.

### Study Procedures

During July–October 2015, we screened serum samples from study participants by using a spike protein subunit 1 (S1)–based ELISA. To confirm results of ELISA-reactive samples, we used indirect immunofluorescent antibody (IFA) and microneutralization (MN) assays (*14*–*16*). For MERS-CoV patients with a nonreactive ELISA result, we collected a follow-up sample 14–21 days later for repeat ELISA.

Study participants were considered candidates for plasma donation if they 1) had a reactive ELISA result; 2) had a MN assay titer of >100; 3) had no clinical or laboratory evidence of ongoing MERS-CoV infection; and 4) met the eligibility for plasma donation according to the institutional criteria, which were in accordance with WHO guidelines (*17*). Persons who met all criteria were eligible for plasma donation according to the position paper of the WHO Blood Regulators Network (*18*).

### Outcome Measures

The primary outcome of this first phase of the study was the feasibility of conducting the second phase. Feasibility was measured by our ability to screen and identify a sufficient number of potential plasma donors to provide enough high-titer, fresh-frozen plasma to enroll and provide transfusions to 20 patients over 12 months. Each phase 2–enrolled patient would require 2 fresh-frozen plasma units (250–350 mL/unit).

### Laboratory Procedures

We first conducted testing for MERS-CoV by rRT-PCR. We extracted RNA from respiratory specimens (nasopharyngeal swab, tracheal aspirate, bronchoalveolar lavage) using the MagNA Pure 96 Viral NA Kit (Roche Applied Science, Indianapolis, IN, USA). We tested the extracted nucleic acids by rRT-PCR targeting the upstream envelope protein gene (upE) and open-reading frame 1a (ORF1a) regions of the MERS-CoV genome on a LightCycler 480 System (Roche Diagnostics, Mannheim, Germany) real-time PCR (*19*). A positive control for ORF1a and upE rRT-PCR was performed according to the manufacturer’s instructions. To be consistent with the cutoff used by the Saudi Arabia Ministry of Health reference laboratory, we considered a cycle threshold (C_t_) of <35 the cutoff for upE and ORF1a. For C_t_s >35, we repeated the testing using different samples, preferably from the lower respiratory tract, to avoid false-positive results.

We detected MERS-CoV antibodies by ELISA (Euroimmun AG, Lubeck, Germany), using wells coated with MERS-CoV S1 antigen (*20*,*21*). Serum samples were diluted (1:100) and incubated with antigens according to the ELISA manufacturer’s instructions. Positive and negative control serum and calibration samples were included. Antibodies were detected by adding peroxidase-labeled rabbit anti–human IgG (Euroimmun AG, Lubeck). Results were reported as the optical density (OD) ratio, which was calculated as the OD value of the patient’s sample divided by the calibrator OD value. We used cutoff values recommended by the ELISA kit manufacturer: a ratio of <0.8 was considered negative, >0.8 to <1.1 was considered borderline, and >1.1 was considered positive.

We used an IFA (Euroimmun AG) according to the manufacturer’s instructions to detect MERS-CoV antibodies. Serum samples were diluted in doubling dilutions, starting with 1:10 and ending with 1:1,280, in sample buffer and then incubated with Vero B4 cells infected with HCoV-EMC (Euroimmun AG). MERS-CoV IgG was detected by adding FITC-labeled goat anti–human IgG (Euroimmun AG); positive and negative controls were included. Samples with an IFA titer of >1:10 were considered reactive according to the IFA manufacturer’s instructions. Our original protocol used an IFA cutoff of >160 to define suitable donors for plasma (*13*). In the course of the study, MN became available, and we revised the criteria for plasma donation to be based on MN assay results.

The presence of neutralizing MERS-CoV antibodies was also assessed using a MN assay (*16*). In brief, 2 × 10^4^ Vero cells/well were plated onto a 96-well microtiter plate. After 24 h, 2-fold serial dilutions of serum samples (heat-inactivated at 56°C for 30 min) were incubated with an equal volume of the MERS-CoV strain Jordan-N3/2012 (200 TCID_50_ [50% tissue culture infectious doses]) for 1 h at 37°C (*16*). Medium was aspirated from the microtiter plate, and 200 μL of the serum–virus mixture was added to the wells in triplicate. The plate was incubated for 48 h at 37°C in a humidified chamber with 5% carbon dioxide, after which the serum–virus mixture was aspirated and the cells were fixed by adding 100 μL of a 1:1 mixture of cold ethanol and methanol. The plate was then incubated at −80°C for 30 min, washed 5 times with PBS, and processed as described above for ELISA, using rabbit anti–coronavirus spike protein antibody and horseradish peroxidase–conjugated goat anti–rabbit IgG secondary antibody. Plates were developed using ABTS substrate (KPL Inc., Gaithersburg, MD, USA); OD was measured at 405 nm. Controls consisted of uninfected cells and cells infected with 200 TCID_50_ of MERS-CoV. The highest dilution of serum sample that resulted in a >50% reduction in OD, compared with the control containing no antibody, was reported as the 50% virus neutralization titer.

rRT-PCR, ELISA, and IFA testing for MERS-CoV were performed at the King Abdulaziz Medical City laboratory. MN was performed at the Naval Medical Research Center (Silver Spring, MD, USA).

### Statistical Analysis

We used descriptive statistics (i.e., numbers and proportions, means ± SD, and medians with quartile 1 [Q1] and Q3 values) for measurements for eligible donors and participants with seroreactive test results. We used the Pearson correlation to test for correlations between ELISA OD and IFA and MN titers.

### Ethical Considerations

The identity of study participants with MERS-CoV was known only to investigators listed on the approved King Abdullah International Medical Research Center protocol. All samples were delinked from any identifiable personal information when provided to nonlisted investigators.

## Results

### Serologic Findings for Healthcare Workers

**Figure 1:**
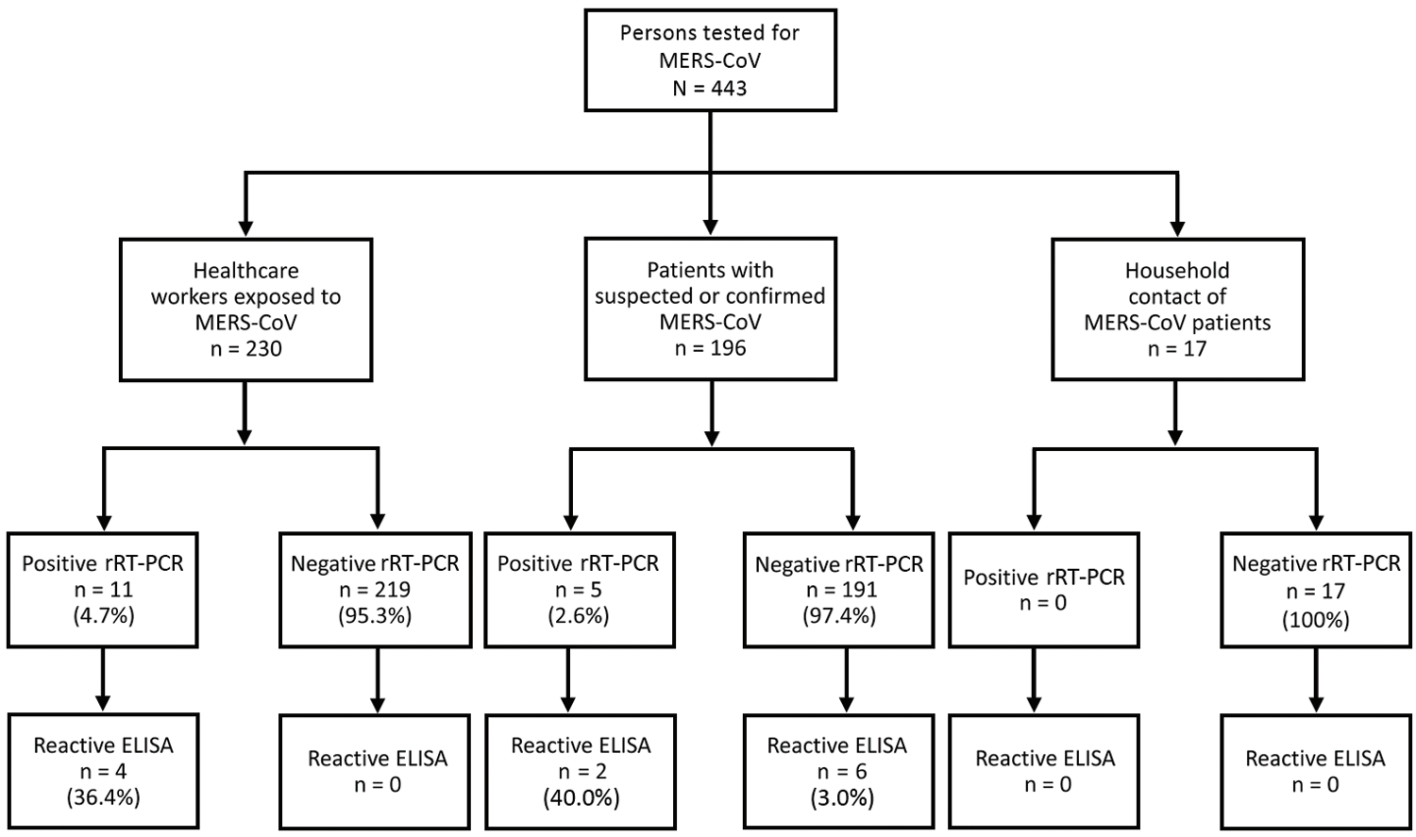
Antibody test results for 443 persons in a study determining the feasibility of using convalescent plasma immunotherapy for Middle East respiratory coronavirus (MERS-CoV) infection, Saudi Arabia. rRT-PCR, real-time reverse transcription PCR.

We contacted 692 healthcare workers who had a history of exposure to or a diagnosis of MERS-CoV infection. Of those 692 healthcare workers, 230 (33%) consented to serum sampling and were tested (Table 1); 11 had a history of laboratory-confirmed MERS-CoV infection, and 219 had a history of exposure but were MERS-CoV rRT-PCR negative during their asymptomatic or potential immediate postincubation period. Only 4 (36.7%) of 11 healthcare workers who had a history of laboratory-confirmed MERS-CoV infection had ELISA-reactive serum samples after a median of 381 days (Q1 246 days, Q3 485 days) after infection (Figure 1). The confirmatory IFA was reactive for all 4 of those healthcare workers, and MN was reactive for 3 (Table 2). However, only 1 healthcare worker (participant no. 9) had a high MN titer (800) (Table 2), but she was not considered a candidate for plasma donation because of a previous pregnancy. Exposed healthcare workers who had negative MERS-CoV rRT-PCR results also had nonreactive ELISA results.

**Table 1:** Characteristics of participants in a study for the feasibility of collecting convalescent plasma from persons who had been infected with or exposed to MERS-CoV, Saudi Arabia, July–October 2015*

Characteristic	Value
Healthcare workers exposed to laboratory-confirmed MERS-CoV patients, N = 230	
Median age, y (Q1, Q3)	35 (29, 42)
Sex	
M	34 (14.8)
F	196 (85.2)
Work-associated exposure	
Intubation	52 (22.6)
Bronchoscopy	22 (9.6)
Tracheal suctioning or inhalation therapy	72 (31.3)
Patient care	117 (50.9)
Reported total duration of exposure†	
<24 h	66/199 (33.2)
>24 h	133/199 (66.8)
Reported exposure intensity‡	
Mild	108/200 (54.0)
Moderate	60/200 (30.0)
Severe	31/200 (15.5)
Laboratory-confirmed MERS-CoV infection	11 (4.8)
ELISA-reactive serum sample	4 (1.7)
Median time from exposure to testing positive, d (Q1, Q3)	381 (246, 485)
Patients with suspected or laboratory-confirmed MERS-CoV infection, N = 196	
Median age, y (Q1, Q3)	65 (49, 76)
Sex	
M	97 (49.5)
F	99 (50.5)
Hospitalization admission area	
Intensive care unit	11 (5.8)
Emergency room	183 (88.8)
Ward	2 (0.97)
Laboratory-confirmed MERS-CoV infection	5 (2.6)
ELISA-reactive serum sample	8 (4.1)
Median time to testing positive, d (Q1, Q3)	7 (4, 12)
Household contacts of confirmed MERS-CoV patients, N = 17	
Median age (range), y	37 (26, 46)
Sex	
M	6 (35.3)
F	11 (64.7)
Laboratory-confirmed MERS-CoV infection	0
ELISA-reactive serum sample	0
Median time to antibody testing, d (Q1, Q3)	34 (34, 34)

*Unless otherwise specified, data are no. (%). Q1 and Q3, quartiles 1 and 3, respectively; MERS-CoV, Middle East respiratory syndrome coronavirus. †Data from a self-administered survey question answered by 199 healthcare workers. ‡Data from a self-administered survey question answered by 200 healthcare workers.

**Table 2:** Characteristics and findings for participants with MERS-CoV antibodies detected by ELISA in a study determining the feasibility of using convalescent plasma immunotherapy for MERS-CoV infection, Saudi Arabia*

Participant no.	Age, y/sex	Symptom at first medical visit	Admitted to ICU	MERS-CoV rRT-PCR	Days from symptom onset or exposure to serum sampling	OD ratio	IFA	MN titer
Patient								
1	70/M	ARI	No	−	35	2.00	1:10	100
2	61/F	ARI	No	+	10	1.12	Nonreactive	200
3	40/F	ARI	No	−	4	3.66	1:20	100
4	63/M	ARI	No	−	27	3.95	1:80	200
5	76/M	ARI	No	+	13	2.59	1:20	200
6	73/M	ARI	No	−	4	1.62	Nonreactive	Nonreactive
7	69/M	ARI	Yes	+	87	4.70	1:1,280	400†
8	71/M	ARI	No	−	9	1.86	Nonreactive	Nonreactive
Healthcare worker								
9	46/F	ARI	Yes	+	24	5.51	1:40	800
10	27/M	None	Yes	+	273	2.33	1:20	50
11	31/M	ARI	No	+	365	1.46	1:10	Nonreactive
12	33/F	None	No	+	365	2.34	1:10	50

**Figure 2:**
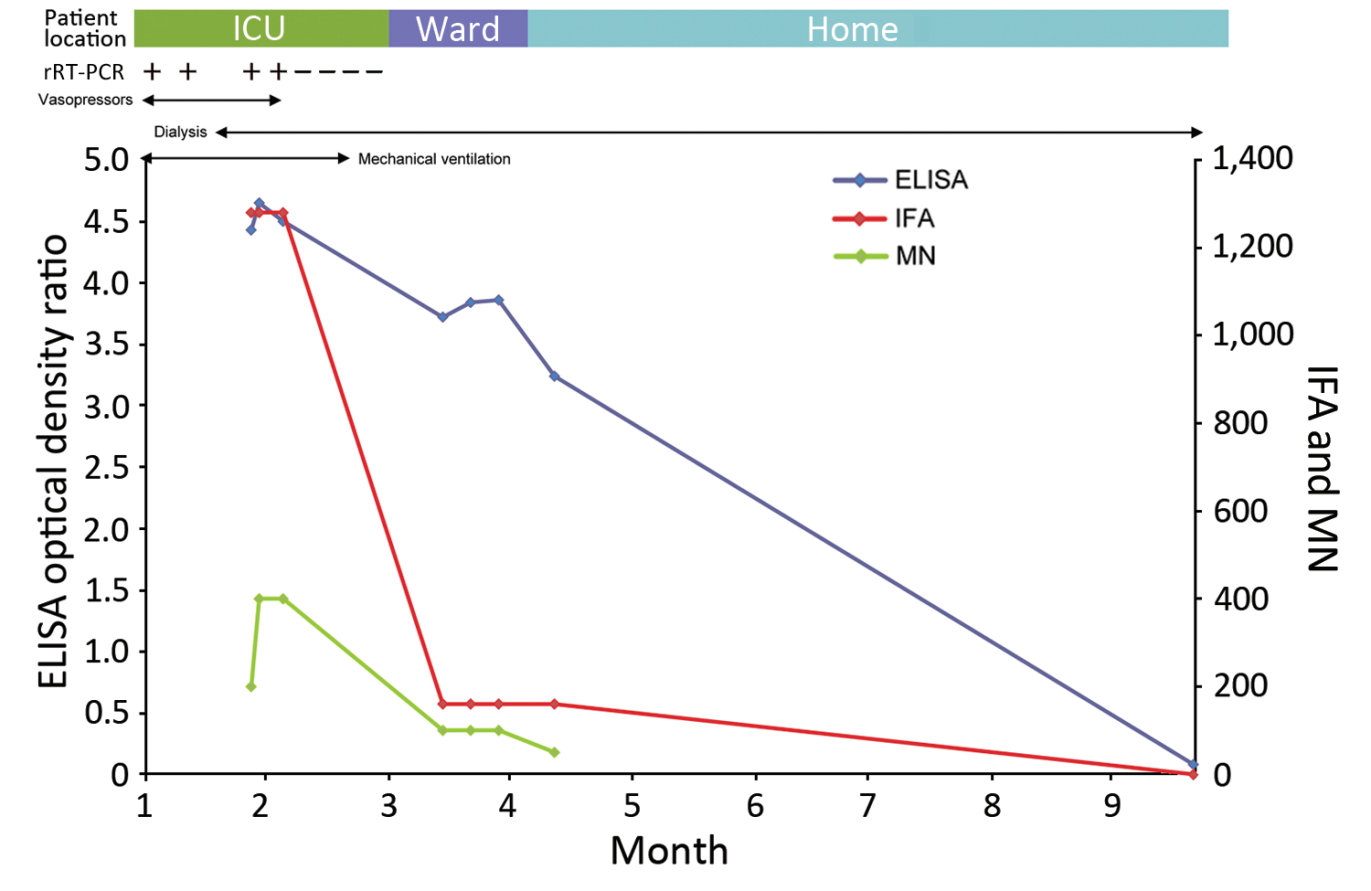
Clinical and laboratory timeline for a Middle East respiratory coronavirus–infected patient with high ELISA, indirect immunofluorescent antibody (IFA), and microneutralization (MN) titers. The highest titers were measured while the patient had active infection and was critically ill. The ELISA optical density ratio and IFA and MN titers declined as the patient recovered. ICU, intensive care unit; rRT-PCR, real-time reverse transcription PCR; ward, hospital ward; −, negative; +, positive.

*ARI, acute respiratory infection; ICU, intensive care unit; IFA, indirect immunofluorescent antibody; OD ratio, optical density value of patient sample/optical density value of calibrator; MERS-CoV, Middle East respiratory syndrome coronavirus; MN, microneutralization assay; rRT-PCR, real-time reverse transcription PCR; −, negative; +, positive. †Serial tests were performed for the patient (Figure 2). Values shown are the highest values for the patient.

### Serologic Findings for Patients with Suspected or Laboratory-Confirmed MERS-CoV Infection

A total of 196 patients with suspected or laboratory-confirmed MERS-CoV infection were tested; 183 (88.8%) were hospitalized in the emergency department, 11 (5.8%) in the intensive care unit, and 2 (0.97%) in the medical wards (Table 1). Two (40%) of 5 patients with laboratory-confirmed MERS-CoV and 6 (3%) of 191 who were MERS-CoV rRT-PCR negative had ELISA-reactive serum samples. 

IFA and MN assay results were positive for 6 (75%) of 8 patients who had ELISA-reactive serum samples; the 2 patients who had nonreactive IFA results also had nonreactive MN results. One of the 6 patients (no. 7) had high IFA (1:1,280) and MN (400) titers (Table 2). This patient, a 69-year-old man, was admitted to the intensive care unit with MERS-CoV infection resulting in acute respiratory distress syndrome, acute kidney injury, and shock. He required mechanical ventilation, renal replacement therapy, and vasopressors (Figure 2). The high titer occurred while he was in intensive care, 32 days after symptom onset. His serologic titers by ELISA, IFA, and MN declined progressively as he recovered clinically; ELISA and IFA were nonreactive by 8 months after hospital admission (Figure 2). Of the 6 patients, 5 (nos. 1–5) had MN titers >100 (Table 2), but these patients did not meet clinical criteria for plasma donation because of age, concurrent conditions, or previous pregnancy.

Of note, 3 patients with laboratory-confirmed MERS-CoV infection had a nonreactive ELISA; these 3 samples were collected 3, 6, and 36 days after symptom onset. Two of the patients died before the test was repeated. For the third patient, repeat ELISAs at 2 and 4 weeks after the first nonreactive ELISA were negative.

### Serologic Findings for Household Contacts

A median of 34 days (Q1 34 days, Q3 34 days) after 2 patients received a laboratory diagnosis of MERS-CoV infection, we tested 3 household contacts for 1 of the patients and 14 for the other (Table 1). Serum samples for all 17 contacts were nonreactive by ELISA (Figure 1; Table 2).

### Correlation of ELISA, IFA and MN Titers

**Figure 3:**
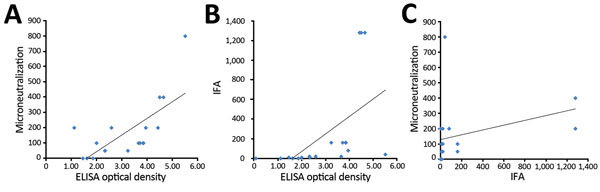
Correlation between ELISA optical density and antibody assay results in a study determining the feasibility of using convalescent plasma immunotherapy for Middle East respiratory coronavirus infection, Saudi Arabia. A) Correlation between ELISA and microneutralization assay results (Pearson correlation coefficient 0.70, p = 0.001). B) Correlation between ELISA and indirect immunofluorescent antibody (IFA) assay results (Pearson correlation coefficient 0.55, p = 0.015). C) Correlation between IFA and microneutralization assay results. (Pearson correlation coefficient 0.38, p = 0.12).

ELISA and MN results were highly correlated (Pearson correlation coefficient 0.70, p = 0.001) (Figure 3). However, ELISA and IFA results showed only a modest correlation (Pearson correlation coefficient 0.55, p = 0.015), and IFA and MN results were not statistically significantly correlated (Pearson correlation coefficient 0.38, p = 0.12).

## Discussion

Our results indicate that it would be possible to obtain quantities of convalescent plasma large enough to use in therapeutic studies or in a large number of MERS-CoV patients; however, large-scale screening would be required because of the limited availability of eligible potential donors with sufficient levels of antibody. Our findings suggest that recently recovered MERS patients may be suitable potential donors, provided they meet other plasma donation criteria. Of note, none of the seropositive persons in our study met our clinical and laboratory criteria for plasma donation.

Our findings show that serum antibody to MERS-CoV was infrequently reactive by ELISA; however, reactivity may have been affected by the timing of sample collection or severity of the illness. Most of the small subset of participants with ELISA-reactive serum samples had MERS-CoV antibodies as assessed by IFA and MN. ELISA results and MN titers were highly correlated; IFA and MN were not. One healthcare worker had high MN titers, but she did not meet the clinical criteria for plasma donation. Another critically ill patient had high antibody titers by the 3 assays, but antibody titers declined quickly as the patient recovered clinically, and he was not eligible to donate plasma.

In accordance with WHO and US Centers for Diseases Control and Prevention guidelines, we used ELISA to screen for MERS-CoV IgG and IFA and MN assays to confirm positive results (*14*,*15*). The ELISA is based on the virus S1 protein as antigen, and the IFA is based on detection of virus-specific antibodies, using cell cultures infected with the virus. The MERS-CoV spike protein is a glycoprotein that forms the spikes of the virus, and the N terminal component (S1) is believed responsible for the first step of virus entry into the host cell (*22*). Our original protocol used IFA as a confirmatory test; however, we switched to MN when that assay became available. Our findings showed a high correlation between ELISA and MN results but not between IFA and MN results, which may indicate that MN is a better confirmatory test. However, for 1 patient in our study, the 3 tests showed similar results (Figure 2), and other studies have shown good correlation between the tests (*20*), which may indicate that the lack of correlation shown between IFA and MN in our study was associated with sample size.

Our findings suggest that the low prevalence of seroreactivity for MERS-CoV, even among persons with confirmed or suspected infection, may be a reflection of a short-lasting antibody response. It is possible that some of the study participants were seronegative at the time of testing because the window for positive serologic results had passed. A short-lasting immune response may also partly explain why negligible or low levels of MERS-CoV seroreactivity have been detected in persons at risk for the disease (i.e., camel and abattoir workers) in Saudi Arabia and elsewhere (*23*–*26*). A seroprevalence study conducted during December 2012–December 2013 showed MERS-CoV antibodies in only 0.15% of the general population (n = 10,009) in all 13 provinces in Saudi Arabia (*21*). Seroprevalence was also low among camel shepherds (2.3%, n = 87) and slaughterhouse workers (3.6%, n = 140), albeit higher than in the general population (*21*). The clinical relevance of antibody titers in protecting against subsequent MERS-CoV infection is uncertain.

Similar findings have been described with other coronaviruses. Cao et al. (*27*) studied specific and neutralizing antibody titers in 56 patients who recovered from SARS-CoV infection. Their findings showed that SARS-CoV IgG and neutralizing antibodies peaked at 4 months and then began diminishing, reaching undetectable levels in 25.6% (IgG) and 16.1% (neutralizing antibodies) of patients at 36 months. Xie et al. (*28*) showed that SARS-CoV IgG decreased over 1 year in recovering SARS patients. In an experiment of intranasal inoculation of CoV 229E in human volunteers, Callow et al. (*29*) studied the time course of specific antibody response and found that those antibodies peaked 1 week after the inoculation and then began declining. Furthermore, it appears that the antibody immune response to MERS-CoV in humans differs from that in camels. Alagaili et al. (*30*) showed that 74% of 150 camels from different parts of Saudi Arabia have antibodies to MERS-CoV by ELISA, and the prevalence of antibodies is higher in older camels (95%).

Two patients in the 2015 MERS-CoV outbreak in South Korea were reported to have received convalescent plasma collected from recovered patients (*31*). It is unclear whether the plasma was tested for the presence of MERS-CoV antibodies. Our study demonstrates that such testing should be mandatory for donated convalescent plasma because of the low prevalence of MERS-CoV antibodies, even in patients with past laboratory-confirmed MERS-CoV infection. Without such testing, the presence of antibodies to MERS-CoV cannot be confirmed, and the convalescent plasma may not be associated with a protective effect. Our study also highlights the need for prospective serology studies to better understand the humoral response to MERS-CoV infection.

The strengths of our study are that we screened a large number of persons, including patients with laboratory-confirmed MERS-CoV infection, and used screening and confirmatory antibody assays. A study limitation was the small number of household contacts who were screened, although, based on our findings and those of others (*20*), a small proportion of household contacts are likely to show seroreactivity. Only one third of invited healthcare workers participated in the study. We used an S1-based ELISA for screening, and our study did not address the magnitude and duration of other antibody isotypes in the immune response. The interval between illness and recovery was prolonged in most of the exposed healthcare workers (median interval >1 year). It is possible that earlier sampling would have resulted in the detection of more reactivity and higher antibody titers. Our study, which was designed to screen for antibodies in convalescent plasma, was not designed to characterize the immune response to MERS-CoV infection or to identify clinical correlates of the presence or absence of MERS-CoV antibodies.

Further testing is needed to determine whether the antibodies in convalescent plasma are clinically effective against MERS-CoV infection. Our findings suggest the need to explore other passive immunotherapeutic approaches, such as monoclonal or polyclonal human antibodies from transchromosomic bovines (*16*,*32*) and, possibly, polyclonal antibodies from camels (*12*). Our findings also raise questions about whether naturally occurring infections and potential MERS-CoV vaccines will offer long-lasting immunity.
